# Quantum Crystallography: the 100-year revolution

**DOI:** 10.1107/S2052252524007164

**Published:** 2025-10-27

**Authors:** Malcolm J. Cooper

**Affiliations:** a Department of Physics University of Warwick CoventryCV4 7AL United Kingdom

**Keywords:** quantum crystallography, Compton scattering, electron density

## Abstract

The IUCr’s close association with quantum crystallography, rightly celebrated by this focused anniversary issue, has its origins in regular meetings of experimentalists and theoreticians interested in electron charge and spin density back in the 1960s. That *ad hoc* group was adopted by the IUCr in the 1970s, when a Commission was created and latterly it has been renamed as the Commission on Quantum Crystallography. The history is summarized in this foreword to the issue.

Those who are passionate about the history of science will debate long and hard about the time and place of the birth of quantum mechanics, but going back exactly one century one finds that 1925 is surely one of the most significant years of that tumultuous decade, marking, as it did, Schrödinger’s development of wave mechanics and the contributions of Heisenberg, Born and Jordan to matrix mechanics. Nevertheless, as we now recognize, there was much published evidence before that year of the failure of classical physics to explain various phenomena, such as the spectrum of black-body radiation, the photoelectric effect and indeed the nature of radiation itself (corpuscular or wave-like), and much evidence that a quantum explanation was needed. To my mind, a turning point was two years earlier, in 1923, in the elucidation of what we now know as the Compton effect. Prior to Compton’s own studies other scientists, such as Florance and especially Gray, had shown unequivocally that there was a decrease in the ‘quality’ (*i.e.* penetrating power) of radiation when scattered, in other words, attributing a wave nature to the radiation, the wavelength had increased and the frequency had dropped. This was a conclusion so obviously counter to classical physics, which required scattering to be elastic, that it was virtually completely ignored or even ridiculed. Even Compton, a diehard classical physicist, only reluctantly adopted the quantum equations for the conservation of energy and momentum in treating the scattering event as a ‘billiard ball’ collision between photon and electron as a convenient last resort to explain his X-ray data: he did not really believe in it. Nonetheless, Compton’s beautiful X-ray experiments were pivotal in giving confidence in the espousal of quantum mechanics by increasing cohorts of scientists in the following years.

It is therefore fitting that the current IUCr Commission on Quantum Crystallography had its origin in the pursuit of Compton scattering as well as X-ray diffraction, back in the 1960s. That was largely down to one individual, Dick Weiss, who worked at the US Army Materials Labs at Watertown Arsenal, in Massachusetts, USA. As an X-ray and neutron diffractionist he was interested in the electron density distribution in transition metals and particularly interested in the behaviour of the 3*d* electrons in Fe. However, unbeknown to him, his single-crystal diffraction X-ray results on Fe were plagued by extinction problems and his misplaced conclusions about the electron distribution were rejected by the research community. Dick Weiss’s response to this setback was abruptly to change course to Compton scattering studies of electron density. The Doppler-like broadening of the Compton-scattered line results from the motion (momentum) of the target scattering electrons and so electron momentum is measured in such an experiment. A description of a quantum system in terms of its wavefunction in momentum space is just as complete, if not as familiar, as one in position space. Dick seized upon an old 1939 result for Compton scattering from lithium which implied that the velocities/momenta of the conduction electrons were several times larger than Fermi–Dirac statistics would dictate for a monovalent metal. He set out to challenge this old finding, collaborating with myself and John Leake at the Cavendish Laboratory in Cambridge, UK, and together we showed, in 1965, that the electron velocities (momenta) were reassuringly just as quantum mechanics predicted. Thus the study of electron momentum density with X-rays and latterly gamma rays, through the Compton effect, was reborn, albeit four decades after Compton’s work.

As well as studies of electron momentum density, the Quantum Crystallography Commission has inherited another activity from Dick Weiss and his colleagues at Watertown Arsenal, namely the ‘Sagamore’ series of conferences. These aimed to bring together an international array of materials scientists at a remote venue, Sagamore, in Upper New York State on Lake Raquette. They initially focused on electron charge and spin densities as revealed by X-ray and neutron diffraction, respectively. The first meeting of what became a triennial conference series was held in 1964 with a second at the same location in 1967. Sagamore meetings were very much in the same vein as present-day Gordon conferences, with plenty of time devoted to unscheduled encounters, discussions by the lakeside, healthy walks in the woods and fiercely contested games of table tennis! Perhaps the significant difference between the Sagamore and Gordon conferences was the fact that in the former the US Army hosted a free bar ‘to aid discussions’; it was available at any time of day or allegedly night for the duration of the meeting! With the conversion of Dick Weiss to the study of electron momentum distributions via Compton scattering, the Sagamore series’ remit was enlarged to encompass ‘Charge, Spin and Momentum Densities’. In acknowledgement of the strong interest in electron density determination in Europe, the Sagamore series next went walkabout outside the USA with a meeting in Aussios, France, in 1970, under the chairmanship of Felix Bertaut, from Grenoble, and in 1973 in Minsk (then in Russia), organized by Nikolai Sirota. The American army’s free bar survived as far as Aussios but proved impossible in Russia!

It was at about this time that Dick Weiss, Felix Bertaut and I thought that the Sagamore organization should become less *adhoc* and have a permanent home by being associated with a respected international parent body. The IUCr was the obvious choice and so the three of us went along to the IUCr’s Amsterdam Congress in 1975 to make the case for a new IUCr commission. Our pleas were successful and an *ad interim* IUCr Commission on Charge, Spin and Momentum Densities was created. The Commission was confirmed as permanent in 1978 at IUCr’s Warsaw congress. It was made clear by the IUCr Executive that running the triennial Sagamore conference series, admirable as that was, was not enough for an active Commission. It should have other scientific projects, and so it did initiate two standardization projects, with the Compton scattering community setting about measuring the Compton profile of water to compare their data-processing routines, while the diffractionists studied a single crystal of oxalic acid with a similar aim. There have been other projects over the years, but it is fair to say that the Sagamore conference series has remained centre stage for the Commission for many decades. In total there have been nineteen Sagamore conferences in twelve different countries over a period of more than half a century, only interrupted by the COVID-19 pandemic. From the outset all have attracted a healthy mixture of leaders in the field (for example two Nobel Prize winners attended Sagamore III in 1970) and many early-career researchers, some supported through IUCr funds.

Despite the impetus provided by Compton scattering’s protagonists in the Commission’s early days, the bulk of the activity, both experimental and theoretical, has always been on charge density in position space, and although momentum-space studies remain as an aspect of the Commission’s work, it would be fair to say that they are somewhat subdued in the present day. Increasingly the Commission has shown the need for quantum descriptions in crystallographic studies, as sticking with the independent atom model (IAM) as the basis for refinement and interpretation was recognized as inadequate. New quantum methodologies, beyond the IAM, allow the determination of accurate and precise electron charge and spin density distributions in position and momentum space from diffraction and scattering experiments, and also provide a wealth of parameters used to characterize the nature of chemical bonding or to quantify physicochemical properties. It is not surprising that the Commission desired to adopt a new name to reflect the increasing emphasis on quantum descriptions. Thus at the Hyderabad Congress, in 2017, the IUCr approved a change of name from Charge, Spin and Momentum Densities to the more succinct, all-embracing, Quantum Crystallography.

So, what of quantum crystallography today? There is a growing recognition, shown in the articles in this collection, that the interpretation of crystal structure is moving to a quantum mechanical basis and vice versa – the calculation of physical and chemical properties must start from an accurate quantum mechanical description of the structure. Evidence for all this comes readily from this anniversary compilation issue, celebrating 100 years of quantum mechanics in crystal structure determination. It contains articles from many leading groups worldwide but with an intriguingly large number based in European countries. It would be interesting to know the reason for these clusters, but there is no doubting the quantity and quality of work falling under the umbrella of quantum crystallography that comes particularly out of France, Germany, Poland and the UK, with other significant contributions from labs within Italy and of course from the USA.

When I look back over my 60 years of involvement in this field, I feel proud of what the IUCr has achieved in first establishing a Commission on Charge, Spin and Momentum Densities and, more recently, endorsing its transformation into the Quantum Crystallography Commission. The work that has been done under the Commission’s auspices is royally celebrated in this series of articles, all of which can be found at https://journals.iucr.org/special_issues/2025/QCr/. Read on!

